# Determination of Best Criteria to Determine Final and Initial Speeds within Ramp Exercise Testing Protocols

**DOI:** 10.1155/2012/542402

**Published:** 2012-11-01

**Authors:** Sidney C. da Silva, Walace D. Monteiro, Felipe A. Cunha, Jonathan Myers, Paulo T. V. Farinatti

**Affiliations:** ^1^Department of Sports Science, Brazilian Olympic Committee, Avenida das Américas 899, 22631-000 Rio de Janeiro, RJ, Brazil; ^2^Laboratory of Physical Activity and Health Promotion, Rio de Janeiro State University, Rua São Francisco Xavier 524, Sala 8121F, 20550-900 Rio de Janeiro, RJ, Brazil; ^3^Graduate Program in Sciences of Physical Activity, Salgado de Oliveira University, Rua Marechal Deodoro 217, No. 2 Andar, 24030-060 Niteroi, RJ, Brazil; ^4^Graduate Program in Medical Sciences, Rio de Janeiro State University, Avenida Professor Manoel de Abreu, 444/No. 2 Andar, Vila Isabel, 20550-170 Rio de Janeiro, RJ, Brazil; ^5^Cardiology Division, Palo Alto VA Health Care System, Cardiology 111C, 3801 Miranda Avenue, Palo Alto, CA 94304, USA

## Abstract

This study compared strategies to define final and initial speeds for designing ramp protocols. *V*_O_2_max _ was directly assessed in 117 subjects (29 ± 8 yrs) and estimated by three nonexercise models: (1) Veterans Specific Activity Questionnaire (VSAQ); (2) Rating of Perceived Capacity (RPC); (3) Questionnaire of Cardiorespiratory Fitness (CRF). Thirty seven subjects (30 ± 9 yrs) performed three additional tests with initial speeds corresponding to 50% of estimated *V*_O_2_max _ and 50% and 60% of measured *V*_O_2_max _. Significant differences (*P* < 0.001) were found between *V*_O_2_max _ measured (41.5 ± 6.6 mL*·*kg^−1^ *·*min^−1^) and estimated by VSAQ (36.6 ± 6.6 mL*·*kg^−1^ *·*min^−1^) and CRF (45.0 ± 5.3 mL*·*kg^−1^ *·*min^−1^), but not RPC (41.3 ± 6.2 mL*·*kg^−1^ *·*min^−1^). The CRF had the highest ICC, the lowest SEE, and better limits of agreement with *V*_O_2_max _ compared to the other instruments. Initial speeds from 50%–60% *V*_O_2_max _ estimated by CRF or measured produced similar *V*_O_2_max _ (40.7 ± 5.9; 40.0 ± 5.6; 40.3 ± 5.5 mL*·*kg^−1^ *·*min^−1^ resp., *P* = 0.14). The closest relationship to identity line was found in tests beginning at 50% *V*_O_2_max _ estimated by CRF. In conclusion, CRF was the best option to estimate *V*_O_2_max _ and therefore to define the final speed for ramp protocols. The measured *V*_O_2_max _ was independent of initial speeds, but speeds higher than 50% *V*_O_2_max _ produced poorer submaximal relationships between workload and *V*_O_2__.

## 1. Introduction

Exercise capacity is an independent predictor of risk for cardiovascular disease and mortality among asymptomatic and symptomatic individuals [1–3]. Hence the determination of maximal oxygen uptake (*V*_O_2_max⁡_) is considered to be one of the most important health-related parameters and has been widely used to evaluate cardiorespiratory fitness in health and illness [4–7]. 

However, the determination of exercise capacity is closely related to the test protocol employed [8]. An extensive body of evidence has shown that ramp exercise protocols offer advantages over traditional protocols, because the increase in external work occurs in a constant and continuous fashion, and when designing the protocol the rate of increase in workload can be individualized by a previous estimate of maximal exercise capacity [7, 9–12]. This is associated with greater linearity between *V*_O_2__ and work rate compared to traditional protocols with large and disproportionate work rate increments [9, 11, 13]. Moreover, ramp protocols induce more uniform hemodynamic and respiratory responses, facilitating the acquisition of information at submaximal intensities, such as the ventilatory threshold [9, 13].

Despite the apparent advantages over traditional exercise testing, standardized criteria to guide the application of ramp protocols remain sparse. For instance, a limitation of ramp protocols is the requirement to estimate maximal exercise capacity from an activity scale and then adjust the ramp rate accordingly [14]. In practical terms, an underestimation of maximal exercise capacity will result in a prolonged total test duration, while an overestimation will result in premature test termination and, therefore, inappropriate test protocol for eliciting a true *V*_O_2_max⁡_ [15]. However, there is no consensus in the literature concerning this issue. Available recommendations are generally vague and largely limited to the premise that tests should last between 8 and 12 min [4, 7, 14–17]. The same occurs with regard to the initial work rate of the test—actually we could not find recommendations of standard procedures for its determination [18].

 Thus, the first objective of the present study was to compare three nonexercise models to predict maximal exercise capacity as criteria to determine the final speed of maximal treadmill ramp protocols. A second purpose was to investigate how different initial speeds calculated from %*V*_O_2_max⁡_ influenced the *V*_O_2_max⁡_ measured in the tests.

## 2. Material and Methods

### 2.1. Subjects

A group of 117 subjects (47 women) aged between 18 and 51 years (mean: 29.1 ± 7.6 yrs), with no previous experience in high performance physical training, volunteered for the study. Exclusion criteria included a clinical diagnosis of any clinical condition that could limit exercise performance and the use of any medication with potential cardiovascular influence. All participants were fully informed about the procedures and potential risks before giving written consent to take part in the study, which was approved by the local Institutional Research Ethics Committee.

### 2.2. Procedures

**Figure 1:**
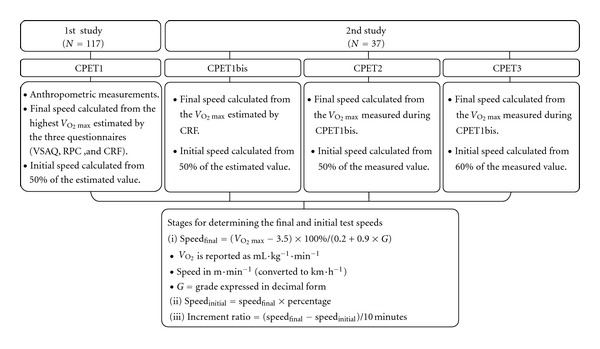
Flowchart of the 1st and 2nd studies including the procedures adopted to determine the workload increments, using nonexercise models to estimate *V*_O_2_max⁡_ and ACSM running equation to calculate the treadmill speeds. *V*_O_2_max⁡_: maximal oxygen uptake; CPET: cardiopulmonary exercise test; VSAQ: Veterans Specific Activity Questionnaire; RPC: Rating of Perceived Capacity; CRF: Questionnaire of Cardiorespiratory Fitness.

A flowchart of the 1st and 2nd studies is presented in Figure 1, detailing the procedures adopted to determine the workload increments using the nonexercise models (1st study—final speed) and different percent *V*_O_2_max⁡_ intensities (2nd study—initial speed).

All 117 subjects enrolled in the first study. After signing the informed consent, the subjects performed the following procedures in a single visit to the laboratory: (a) anthropometric measurements; (b) application of three nonexercise models to estimate *V*_O_2_max⁡_ (Veterans Specific Activity Questionnaire (VSAQ), [19, 20]; Rating of Perceived Capacity (RPC) [21]; Questionnaire of Cardio-respiratory Fitness (CRF) [22]); (c) cardiopulmonary exercise testing. 

The VSAQ was originally developed by Myers et al. [19, 20] with the specific purpose of individualizing ramp protocols. The VSAQ includes a list of physical activities with scores ranging from 1 to 13. The responder indicates which of the listed activities would cause fatigue or shortness of breath. Subjects evaluated in the initial studies with the VSAQ had low cardiorespiratory fitness and a high prevalence of overweight/obesity, hypertension, or coronary disease. Even though further studies have demonstrated that the instrument also provided adequate estimation of *V*_O_2_max⁡_ in healthy active populations [5, 8], there is a lack of research specifically designed to assess its validity within the application of ramp protocols in healthy subjects. The RPC may be considered a variation of the VSAQ [21], presenting different maximal MET levels (ranging from 1 to 20), which are linked to physical activities of several intensities. Subjects rate their perceived capacity by choosing the most strenuous activity they could sustain for 30 min. However, the RPC has been not validated through direct comparison with exercise capacity using cardiopulmonary exercise testing. The CRF was not specifically developed to design ramp protocols, but it has been extensively applied as a nonexercise model to estimate the maximal cardiorespiratory capacity [22]. It is a progressive scale with scores for the intensity of the activities ranging from 0 to 7. The subjects must select the most appropriate score according to the physical activities performed in the last 30 days. The CRF was selected because of the unusual methodological meticulousness applied to its development. A large sample (*N* = 799) of men and women aged 19 to 79 years was tested. The estimated *V*_O_2_max⁡_ was compared to directly measured data, and the questionnaire was cross-validated with another population, which is uncommon in studies assessing such instruments [23, 24]. 

In the first study, the increase in work rate within the cardiopulmonary exercise test (CPET1) was individualized to elicit each subject's limit of tolerance in 10 min, and treadmill grade was set at 0%. Final and initial speeds were determined using ACSM equations for treadmill running [7], considering the intensities corresponding to the highest *V*_O_2_max⁡_ estimated by the non-exercise models (final speed) and 50% of this value (initial speed). The choice of 50% of the estimated *V*_O_2_max⁡_ to determine the initial speed was based on a previous pilot study involving 35 subjects. In this pilot study, the initial speed was set at 1/3 of the estimated *V*_O_2_max⁡_, which corresponded to a mean speed of 4.3 km·h^−1^ and a work rate increase of 0.88 km·h^−1^ each minute. The protocols lasted approximately 12 min (11.3 ± 2.2 min) and subjects remained walking, for about 4 min. Thus, an intensity of 50% *V*_O_2_max⁡_ would probably shorten the test and increase the time in which the subjects would be actually running.

A subgroup of 37 subjects (17 women; age: 29.1 ± 7.6 yrs) was randomly selected to participate in the second study. These subjects performed three additional cardiopulmonary exercise tests, separated by 72 to 120 h intervals. The increase in work rate and treadmill grade were the same applied in CPET1. In the first test (CPET1bis), the final speed was determined using the best non-exercise model as defined in the first study, and the initial speed set at 50% of this value. The other tests (CPET2 and CPET3) were then performed using the results of CPET1bis as reference. In brief, the final speed in CPET1bis was estimated from the maximal exercise capacity provided by CRF, whereas in both CPET2 and CPET3 it corresponded to the speed associated with the *V*_O_2_max⁡_ assessed in CPET1bis. The initial speeds corresponded to 50% *V*_O_2_max⁡_ estimated (CPET1bis), 50% *V*_O_2_max⁡_ measured (CPET2), and 60% *V*_O_2_max⁡_ measured (CPET3). This approach allowed to observe whether initial speeds ranging from 50 to 60% *V*_O_2_max⁡_ (estimated or measured) influenced the results of the tests. 

In the first study the CPET1 was applied by a researcher blinded for the results of the non-exercise models. In the second study, the sequence of tests was defined by a counterbalanced crossover design. The participants were blinded for the %*V*_O_2__ used to establish the initial speeds, and the evaluator was blinded for the purposes of the study. 

The cardiopulmonary exercise test protocols were performed using a super-ATL treadmill (Inbramed, Florianopolis, SC, Brazil), and *V*_O_2__ was averaged and recorded every 30 s. The 30 s time average provided a good compromise between removing noise from *V*_O_2__ data while maintaining the underlying trend [25]. Data was assessed using a mouthpiece and noseclip. Gas exchange was assessed using a VO2000 analyzer (Medical Graphics, Saint Louis, MO, USA), which was calibrated with a certified standard mixture of oxygen (17.01%) and carbon dioxide (5.00%), balanced with nitrogen. The flows and volumes for the pneumotachograph were calibrated with a 3 L syringe (Hans Rudolph, Kansas, MO, USA). Heart rate was monitored using a Polar S-810 device (Polar, Kempele, Finland). Mean ambient temperature and relative humidity during testing were 22.4 ± 1.8°C (range 18–23) and 62.5 ± 4.1% (range 50–75%), respectively.

The criteria for test interruption followed the recommendations of the American College of Sports Medicine [7]. The test was considered to achieve peak capacity when at least three of the following criteria were observed [26]: (a) maximum voluntary exhaustion as reflected by a score of 10 on the Borg CR-10 scale; (b) ≥95% predicted HR max (220—age) or presence of an HR plateau (ΔHR between two consecutive work rates ≤4 beats·min^−1^); (c) presence of a *V*_O_2__ plateau (Δ*V*_O_2__ between two consecutive work rates <2.1 mL·kg^−1^·min^−1^); (d) respiratory exchange ratio > 1.15. Participants were verbally encouraged to achieve maximal effort. Holding onto the side or front rails of the treadmill was not permitted.

### 2.3. Statistical Analyses

Data normality was confirmed by univariate analysis. Therefore the intraclass correlation coefficient (ICC) was used to verify the concordance between the *V*_O_2_max⁡_ assessed in CPET1 and the *V*_O_2_max⁡_ estimated by the non-exercise models. Limits of agreement and bias for measured and estimated *V*_O_2_max⁡_ were determined according to the Bland and Altman method [27]. Intraclass correlation (ICC), *R*-square coefficients (*r* ^2^), and standard errors of estimate (SEE) between actual and estimated *V*_O_2_max⁡_ were also calculated. 

The *V*_O_2_max⁡_ values obtained in CPET1bis, CPET2, and CPET3 were compared by repeated measures ANOVA. Additionally, linear regression was performed for each subject on each protocol in order to compare the relationships between workload and *V*_O_2__, considering data in every 30 s of exercise. Mean ± SD values of intercepts and slopes were determined for each linear regression model. Student *t*-tests for paired samples were used to test whether the intercepts and slopes were significantly different from 0 and 1, respectively [12], and to test possible differences between the regression lines, as described in detail elsewhere [28]. The *r* ^2^ and SEE for the regression models obtained in all tests were calculated as supplementary criteria to define the best initial speed. Two-tailed statistical significance for all tests was accepted as *P* ≤ 0.05. All statistical analyses were performed using Statistica 7.0 (Statsoft, Tulsa, OK, USA) and SPSS 8.0 (IBM, Chicago, IL, USA) statistical analysis software.

## 3. Results

An achieved statistical power of 0.96 for an effect size of 0.25 was obtained by performing a post hoc power analysis (GPower version 3.0.10, Kiel, University of Kiel, Germany) based on the sample size, *P* value, number of repeated measures, and groups.  Table 1 presents the characteristics of the samples comparing strategies to define final and initial speeds.  Table 2 presents values for the assessed *V*_O_2_max⁡_ (mL·kg^−1^·min^−1^) by age and sex groups. 

In the first study, mean duration of CPET1 was 13.3 ± 2.1 min for initial and final speeds of 5.9 ± 0.9 km·h^−1^ and 14.7 ± 2.1 km·h^−1^, respectively. Significant differences were detected between *V*_O_2_max⁡_ assessed in CPET1 (41.5 ± 6.6 mL·kg^−1^·min^−1^) and *V*_O_2_max⁡_ estimated from VSAQ and CRF (*V*_O_2_max⁡_ VSAQ = 36.6 ± 6.6 mL·kg^−1^·min^−1^, *P* < 0.0001; *V*_O_2_max⁡_ CRF = 45.0 ± 5.3 mL·kg^−1^·min^−1^; *P* < 0.0001), but not from RPC (*V*_O_2_max⁡_  RPC = 41.3 ± 6.2 mL·kg^−1^·min^−1^, *P* = 0.99). 

**Figure 2:**
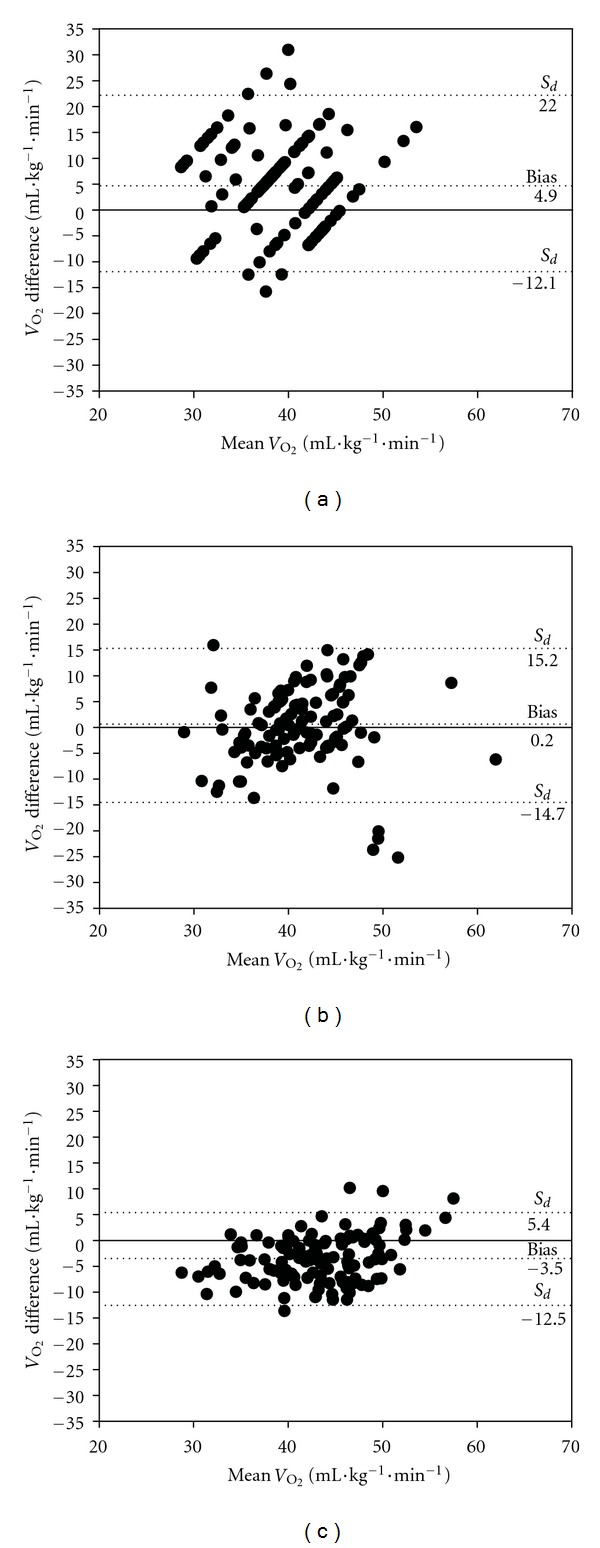
Bland-Altman plot for the individual differences between *V*_O_2_max⁡_ assessed in CPET1 and *V*_O_2_max⁡_ estimated by VSAQ (a), RPC (b), and CRF (c). The first and third horizontal dashed lines in each graph represent the 95% limits of agreement for VSAQ, RPC, CRF, and VSAQ, corresponding, respectively, to −12.1 to 22.0 (−29.1 to 53.0%); −14.7 to 15.2 (−35.5 to 36.6%); −12.5 to 5.4 (−30,0% to 13,0%). *S*_*d*_: standard deviation of the differences.

Figure 2 shows the Bland-Altman analysis, including the limits of agreement for estimated and measured *V*_O_2_max⁡_.  Table 3 presents values for *R*-square, SEE, and ICC between *V*_O_2_max⁡_ measured and estimated by the questionnaires. The RPC provided the lowest mean difference between *V*_O_2_max⁡_ directly assessed in CPET1 and estimated from the questionnaires (RPC = 0.24 mL·kg^−1^·min^−1^; CRF = −3.54 mL·kg^−1^·min^−1^; VSAQ = 4.94 mL·kg^−1^·min^−1^; *P* = 0.05). However, the CRF exhibited better limits of agreement compared to the other instruments. The higher values obtained for CRF with regard to *R*-square and ICC were consistent with the results of the Bland-Altman analysis. The SEE between assessed and estimated *V*_O_2_max⁡_ was also lower in CRF compared to VSAQ and RPC. 

Table 4 shows the distribution of *V*_O_2_max⁡_ assessed in CPET1 according to tertiles, as well the percent agreement between estimated and measured *V*_O_2_max⁡_ in each tertile. The nonparametric Kendall's tau-b correlation between tertiles was similar across the three questionnaires and measured *V*_O_2_max⁡_. However the correlation using the CRF was higher over RPC and VSAQ—the proportion of subjects assigned in the same tertile category was superior for CRF compared to the other questionnaires, and the distribution was more homogeneous. 

With regard to the second study, mean durations of CPET1bis, CPET2, and CPET3 were 13.7 ± 1.8 min, 10.7 ± 1.9 min, and 10.6 ± 0.9 min, respectively. No differences were detected between *V*_O_2_max⁡_ assessed in CPET1bis (used as reference to define final and initial speeds in CPET2 and CPET3), CPET2, and CPET3 (CPET1bis = 40.7 ± 5.9 mL·kg^−1^·min^−1^; CPET2 = 39.8 ± 5.6 mL·kg^−1^·min^−1^; CPET3 = 40.3 ± 5.5 mL·kg^−1^·min^−1^; *P* = 0.142). Mean initial speeds applied in CPET1bis, CPET2, and CPET3 were 5.7 ± 0.8 km·h^−1^, 8.1 ± 0.9 km·h^−1^, and 9.1 ± 1.1 km·h^−1^, respectively.  Table 5 shows the relationships between workload and *V*_O_2__ in the ramp test protocols initiating with speeds corresponding to 50% and 60% *V*_O_2_max⁡_ either measured or estimated (slopes, intercepts, *R*-square, and SEE). CPET1bis showed the closest relationship with the theoretical identity line (slope = 1 and intercept = 0), with the highest *R*-square and lowest SEE in comparison with CPET2 and CPET3. 

## 4. Discussion

The present study aimed to compare different strategies to define final and initial speeds when designing ramp exercise testing protocols for healthy young populations. Three nonexercise models were employed to estimate maximal cardiorespiratory capacity and therefore the final speed. The choice of VSAQ, RPC, and CRF to estimate the *V*_O_2_max⁡_ was due to the fact that these instruments have been frequently applied in previous studies and have been shown to have good potential to estimate the maximal cardiorespiratory capacity in different populations [23, 24]. Two relative intensities (%*V*_O_2_max⁡_) using different initial treadmill speeds were tested.

The values obtained for the *V*_O_2_max⁡_ assessed in CPET1 are consistent with reference values reported by previous research [4, 7, 14, 16]. Our findings on the ICC, *R*-square, SEE, and dispersion in the Bland-Altman plot (see Figure 2) suggest that there are advantages in using the CRF to determine the final speed, in comparison with the other instruments. In contrast, the VSAQ had the poorest precision and highest variability with respect to *V*_O_2_max⁡_ estimation. In their original study, Myers et al. [19] reported a stronger association between estimated and achieved cardiorespiratory capacity over the present data (*r* = 0.79; SEE = 4.97 mL·kg^−1^·min^−1^; *P* = 0.001 versus *r* = 0.40; SEE = 7.63 mL·kg^−1^·min^−1^; *P* = 0.0001, resp.). However, subjects in the two studies differed considerably in terms of clinical and fitness status, which may have contributed to such discrepancy, since poor conditioned individuals are more likely to interrupt earlier the test due to peripheral fatigue. Moreover, Myers et al. [19] did not directly assess the *V*_O_2_max⁡_ in their original research. In a later study, these investigators [20] validated the VSAQ measuring *V*_O_2_max⁡_ directly in a larger sample (*n* = 337). Subjects had similar characteristics as those in the original study, but the results were more similar to our findings (*r* = 0.42; SEE = 9.1 mL·kg^−1^·min^−1^; *P* = 0.001). 

Maeder et al. [5] compared the *V*_O_2_max⁡_ obtained in tests using cycle ergometer and treadmill with the exercise capacity estimated by the VSAQ in healthy subjects. The correlations were similar to our data (cycle ergometer: *r* = 0.46 and treadmill: *r* = 0.50; *P* < 0.0001). More recently, Maeder et al. [8] used the VSAQ to select the optimal treadmill ramp protocol in highly trained individuals and reported a similar correlation between estimated and measured *V*_O_2_max⁡_ (*r* = 0.47), even when using the VSAQ modified nomogram (*r* = 0.56). 

Although the VSAQ was developed to facilitate the individualization of ramp protocols, previous research has not ratified this purpose in all populations. Actually, the available evidence does not support its use in determining the final speed within ramp protocols in healthy and well-conditioned populations. Actually the VSAQ has been shown to be more appropriate to estimate the *V*_O_2_max⁡_ in unfit individuals [20, 29]. The present results confirm this idea. Precision using the VSAQ was lower compared to the other instruments, and the same categorization was obtained in less than 40% of cases. Furthermore, the Bland-Altman plots suggested that in our sample the *V*_O_2_max⁡_ was systematically overestimated by the VSAQ.

The RPC closely paralleled *V*_O_2_max⁡_ assessed in CPET1 (mean difference of 0.24 mL·kg^−1^·min^−1^ or 1%), but exhibited high variability, as evidenced by the Bland-Altman method and SEE (7.60 mL·kg^−1^·min^−1^). This variation accounted for the relatively low ICC and *R*-square values. It is noteworthy that RPC was developed in a sample of 87 young, healthy women (age = 48.4 ± 17.4 years) [21]. However, our experience with this method suggests that strong agreement between estimated and actual *V*_O_2_max⁡_ can be also obtained in men. Interestingly, although our sample consisted of young women (age = 28.2 ± 7.0 years), the comparison between *V*_O_2_max⁡_ directly measured and estimated by RPC showed greater concordance (ICC) and lower variation (SEE) among men versus women (ICC = 0.58 versus 0.42 and SEE = 1.70 mL·kg^−1^·min^−1^ versus 8.35 mL·kg^−1^·min^−1^, resp.). A possible explanation for this is that in the original RPC study the *V*_O_2_max⁡_ was estimated from the work performed on cycle ergometer, and not directly measured. The *V*_O_2_max⁡_ was estimated using maximal work and body mass, assuming as constants the amount of oxygen required for each Watt of power during ramp cycling (10.93 mL·min^−1^·W^−1^) and *V*_O_2__ at rest when sitting on the cycle (4.3 mL·min^−1^). However these unpublished data have been previously determined in a group of healthy men [21], and no information was provided with regard to their possible application in females. 

The CRF has been widely used to estimate maximal cardiorespiratory capacity [12, 30–35]. Although it was not originally developed to help designing ramp protocols, our results indicate that it works well for this purpose. The original study by Matthews et al. [22] showed a higher correlation between *V*_O_2_max⁡_ measured and estimated from CRF than the present study, in a sample of 390 men (*r* = 0.82 versus *r* = 0.61, resp.) and 409 women (*r* = 0.83 versus *r* = 0.69, resp.). However, the SEEs in the total sample (5.7 mL·kg^−1^·min^−1^ versus 5.8 mL·kg^−1^·min^−1^) and in gender subgroups (men: 6.3 mL·kg^−1^·min^−1^ versus 6.0 mL·kg^−1^·min^−1^; women: 5.0 mL·kg^−1^·min^−1^ versus 5.4 mL·kg^−1^·min^−1^) were similar in the two studies. The Bland-Altman analysis showed limits of agreement higher over VSAQ and comparable to RPC, but the CRF had the greatest ICC. In addition, the tertile classifications obtained from CRF were more accurate compared to the other nonexercise models. 

Overall, CRF showed higher concordance with measured *V*_O_2_max⁡_, lower dispersion, and better capacity to discriminate subjects with high and low cardio-respiratory capacity in comparison to VSAQ and RPC. Notably, the CRF may be limited when assessing cardiorespiratory capacity in subjects with *V*_O_2_max⁡_ > 55.0 mL·kg^−1^·min^−1^ [29], which could be a problem when designing ramp protocols in highly fit individuals. However, fewer than 20% of ordinary healthy individuals achieve this level [7]. It therefore seems unlikely that the final speed would be wrongly determined from inaccurate estimation of *V*_O_2_max⁡_ estimation, at least in most healthy nonathletic subjects.

In what concerns the second study, the literature is mixed regarding criteria to determine the initial speed for ramp testing [9, 11]. Recommendations from different expert panels are also ambiguous with regard to this issue [4, 7, 14, 15], and no formal criteria are available on this important aspect of ramp protocols. Our findings suggested that initial speeds within the range corresponding to 50% to 60% *V*_O_2_max⁡_ influenced the duration of the test (CPET1bis = 13.7 ± 1.8 min > CPET2 = 10.7 ± 0.9 min  ≅  CPET3 = 10.6 ± 0.9 min, *P* < 0.0001), but not the achieved *V*_O_2_max⁡_ (CPET1bis = 40.7 ± 5.9 mL·kg^−1^·min^−1^  ≅  CPET2 = 40.0 ± 5.6 mL·kg^−1^·min^−1^  ≅  CPET3 = 40.3 ± 5.5 mL·kg^−1^·min^−1^, *P* = 0.14). From these results, any initial speed within this range would be appropriate for performing ramp tests. In contrast, the relationship between workload and *V*_O_2__ among the tests was affected by the initial speed. Considering the identity line as a reference for the ideal regression between workload and *V*_O_2__, the current results suggest that higher initial speed produced the lowest *R*-squares (e.g., poorest adjustment to the identity line) (CPET3—60% *V*_O_2_max⁡_ < CPET2—50% *V*_O_2_max⁡_ < CPET1bis—50% *V*_O_2_max⁡_).

Early research confirms the concept that the initial speed applied does not influence measured *V*_O_2_max⁡_. Kang et al. compared three incremental treadmill protocols (Åstrand, Bruce, and Costill/Fox) in 25 sedentary subjects (10 women) [36]. The protocols began with speeds of 9.7 km·h^−1^, 2.5 km·h^−1^, and 14.4 km·h^−1^, respectively, and no differences in *V*_O_2_max⁡_ were detected. The relationship between workload and *V*_O_2__ was not specifically addressed, but the authors considered that this could have been good, at least in the Costill/Fox protocol. The high initial speed significantly shortened the tests (to about 5 min) and precluded the identification of the ventilatory threshold. 

In 1991, Myers et al. compared *V*_O_2_max⁡_ obtained during ramp and conventional staged protocols (Bruce and Balke modified), which were very different with regard to the combination of initial speed, treadmill grade, and workload increment. The duration of tests was significantly different (Bruce: 6.6 ± 1.5 min versus Balke: 10.4 ± 3.4 min and Ramp: 9.1 ± 1.4 min, *P* < 0.05), with little impact on *V*_O_2_max⁡_ (Bruce: 22.3 ± 8.0 mL·kg^−1^·min^−1^ versus Balke: 21.1 ± 8.0 mL·kg^−1^·min^−1^ and Ramp: 21.0 ± 8.0 mL·kg^−1^·min^−1^, *P* < 0.05). However, slopes and SEE for the regression curves between workload and *V*_O_2__ showed more linear relationships in the ramp protocol (Bruce: slope = 0.62 and SEE = 4.0 mL·kg^−1^·min^−1^; Balke: Slope = 0.79 and SEE = 3.4 mL·kg^−1^·min^−1^; Ramp: Slope = 0.80 and SEE = 2.5 mL·kg^−1^·min^−1^). In other words, differences in the protocol design may reflect on physiological relationships in submaximal workloads, but not necessarily on the assessed *V*_O_2_max⁡_. Our findings seem to ratify this idea.

In conclusion, CRF was superior in comparison with RPC and VSAQ to estimate maximal cardio-respiratory capacity and should be preferred when attempting to determine an appropriate speed for ramp testing. Initial speeds within the range corresponding to 50–60% *V*_O_2_max⁡_ estimated or measured did not affect assessed *V*_O_2_max⁡_. Nevertheless, speeds higher than 50% *V*_O_2_max⁡_ may influence the quality of submaximal relationships between work rate and *V*_O_2__. Moreover, higher speeds applied at the beginning of ramp protocols may hinder the performance of subjects with poor fitness levels and compromise test results. This information should be considered when data from exercise testing is used to establish relative exercise intensities for exercise prescription.

## Figures and Tables

**Table 1:** Characteristics of the subjects participating in the comparisons regarding the final (*N* = 117) and initial (*N* = 37) speeds.

	Age (years)				Body mass (kg)				Height (cm)				Body fat (%)				*V*_O_2_max⁡_ (mL·kg^−1^·min^−1^)			
	G	M	F	G2	G	M	F	G2	G	M	F	G2	G	M	F	G2	G	M	F	G2
Mean	29.1	29.8	28.2	29.7	71.7	79.7	59.7	72.4	171.2	176.7	163.1	170.4	15.2	11.7	20.4	16.8	41.5	43.9	37.8	40.7
SD	7.6	7.9	7.0	8.6	14.9	12.6	8.8	17.9	9.1	5.9	6.5	10.0	7.0	5.8	5.3	6.5	6.6	5.8	6.1	5.9
Minimum	18	18	19	18	46.7	57.4	46.7	46.5	150.0	163.3	150.0	150.5	2.9	2.9	11.2	6.0	25.6	32.8	25.6	28.5
Maximum	51	47	51	51	92.9	92.9	91.8	90.3	190.0	190.0	176.0	190.0	32.8	27.1	32.8	28.3	61.6	61.6	54.8	52.4

G: total sample (*n* = 117); M: males (*n* = 70); F: females (*n* = 47); G2: subgroup for initial speed comparison (*n* = 37).

**Table 2:** Descriptive values for *V*_O_2_max⁡_ (mL·kg^−1^·min^−1^) by age and sex groups.

	Age (years)					
	Males (*N* = 70)			Females (*N* = 47)		
	18–29 (*N* = 39)	30–39 (*N* = 20)	>40 (*N* = 11)	18–29 (*N* = 32)	30–39 (*N* = 10)	>40 (*N* = 5)
Mean	46.2	41.1	39.4	39.0	34.3	37.0
SD	5.8	4.4	3.9	5.9	6.3	4.6
Minimum	36.5	32.8	34.0	26.2	25.6	29.5
Maximum	61.5	47.9	44.2	54.8	45.3	40.4

**Table 3:** Mean difference (mL·kg^−1^·min^−1^), *R*-square coefficient, standard error of estimate, and intraclass correlation between *V*_O_2_max⁡_ assessed and estimated by three non-exercise models (*N* = 117).

	Total (*N* = 117)					Males (*N* = 70)					Females (*N* = 47)				
	*V*_O_2_max⁡_					*V*_O_2_max⁡_					*V*_O_2_max⁡_				
	Mean difference	*r* ^2^	SEE	ICC	*P*	Mean difference	*r* ^2^	SEE	ICC	*P*	Mean difference	*r* ^2^	SEE	ICC	*P*
VSAQ	4.94 (11.9%)	0.16	7.63	0.57	<0.0001	−1.81 (−4.1%)	0.05	7.92	0.36	<0.0317	−1.24 (−3.3%)	0.07	7.17	0.42	<0.040
RPC	0.24 (1.0%)	0.09	7.60	0.46	<0.001	3.22 (7.3%)	0.17	1.70	0.58	<0.0001	−3.49 (−9.2%)	0.07	8.35	0.42	<0.035
CRF	−3.54 (−8.5%)	0.53	5.75	0.83	<0.0001	−3.89 (−8.9%)	0.37	6.01	0.76	<0.0001	−2.90 (−7.7%)	0.47	5.36	0.81	<0.0001

VSAQ: Veteran Specific Activity Questionnaire using the following equation: *V*_O_2__ (mL·kg^−1^·min^−1^) = (4.7 + 0.97 (VSAQ) − 0.06 (age) × 3.5); for women this value was multiplied by 0.85 [8]; RPC: Rating of Perceived Capacity; CRF: Cardiorespiratory Fitness.

**Table 4:** Percentage of participants ranked in the same tertile, percentage of total agreement, tau-b correlation coefficients between *V*_O_2_max⁡_ measured and estimated by three non-exercise models (VSAQ, RPC, and CRF) (*N* = 117).

	1st Tertile (*n* = 39)	2nd Tertile (*n* = 39)	3rd Tertile (*n* = 39)	Total (*N* = 117)	*R* (tau-b)
*V*_O_2_max⁡_ versus VSAQ	66.66% (26)	5.12% (2)	38.46% (15)	36.75% (43)	0.833
*V*_O_2_max⁡_ versus RPC	43.58% (17)	25.64% (10)	43.58% (17)	37.60% (44)	0.992
*V*_O_2_max⁡_ versus CRF	69.23% (27)	41.02% (16)	58.97% (23)	56.41% (66)	0.983

VSAQ: Veteran Specific Activity Questionnaire; RPC: Rating of Perceived Capacity; CRF: Questionnaire of Cardiorespiratory Fitness.

**Table 5:** Intercept, slope, *R*-square (*r* ^2^), and standard error of estimate (SEE) for the regression models obtained in ramp protocols initiating with speeds corresponding to 50% of the estimated *V*_O_2_max⁡_ (CPET1bis), 50% of the measured *V*_O_2_max⁡_ (CPET2), and 60% of the measure *V*_O_2_max⁡_ (CPET3).

	*Y* intercept	Slope	*r*-Square	SEE (mL·kg^−1^·min^−1^)
*V*_O_2__versus speed in CPET1bis	−4.882 ± 2.696*	0.96 ± 0.027^§^	0.93 ± 0.050	2.14 ± 0.67
*V*_O_2__versus speed in CPET2	−8.270 ± 6.312*	0.94 ± 0.029^§^	0.89 ± 0.054	2.19 ± 0.55
*V*_O_2__versus speed in CPET3	−14.666 ± 8.958*	0.92 ± 0.036^§^	0.86 ± 0.065	2.48 ± 0.67

*Intercept significantly different from zero (*P* < 0.0001).

^§^Slope significantly different from 1.0 (*P* < 0.0001).
